# Sudden Cardiac Arrest in an Adult with Anomalous Origin of the Left Coronary Artery from the Pulmonary Artery (ALCAPA): Case Report

**DOI:** 10.3390/ijerph19031554

**Published:** 2022-01-29

**Authors:** Francesca Romana Prandi, Ali N. Zaidi, Gina LaRocca, Michael Hadley, Maria Riasat, Malcolm O. Anastasius, Pedro R. Moreno, Samin Sharma, Annapoorna Kini, Raghav Murthy, Percy Boateng, Stamatios Lerakis

**Affiliations:** 1 Division of Cardiology, Mount Sinai Heart, Mount Sinai Hospital, Icahn School of Medicine at Mount Sinai, New York, NY 10029, USA; gina.larocca@mountsinai.org (G.L.); michael.hadley@mountsinai.org (M.H.); malcolm.anastasius@mountsinai.org (M.O.A.); pedro.moreno@mountsinai.org (P.R.M.); samin.sharma@mountsinai.org (S.S.); annapoorna.kini@mountsinai.org (A.K.); stamatios.lerakis@mountsinai.org (S.L.); 2 Department of Systems Medicine, Division of Cardiology, University of Rome “Tor Vergata”, 00133 Rome, Italy; 3 Mount Sinai Adult Congenital Heart Disease, Mount Sinai Heart, Icahn School of Medicine at Mount Sinai, New York, NY 10029, USA; ali.zaidi@mountsinai.org; 4 Division of Internal Medicine, Mount Sinai Beth Israel, Icahn School of Medicine at Mount Sinai, New York, NY 10003, USA; maria.riasat@mountsinai.org; 5 Division of Cardiothoracic Surgery, Mount Sinai Heart, Icahn School of Medicine at Mount Sinai, New York, NY 10029, USA; raghav.murthy@mountsinai.org (R.M.); percy.boateng@mountsinai.org (P.B.)

**Keywords:** anomalous origin of coronary artery from pulmonary artery, ALCAPA, cardiac arrest, implantable cardioverter defibrillator, sudden cardiac death, multimodality cardiac imaging

## Abstract

Introduction: Anomalous origin of the left coronary artery from the pulmonary artery (ALCAPA) is a rare coronary artery anomaly that carries 90% mortality in the first year of life when left untreated. The diagnosis of ALCAPA is rare in adulthood, and it includes a broad spectrum of clinical manifestations, including sudden cardiac death (SCD). Case report: We report a rare case of resuscitated sudden cardiac arrest in a 55-year-old female, who was diagnosed with ALCAPA and underwent successful surgical correction and implantable cardioverter defibrillator (ICD) implantation for secondary prevention. Discussion: ALCAPA diagnosis is not confined to childhood, and it represents a rare cause of life-threatening arrhythmias and SCD in the adult population. Surgical correction is recommended, regardless of age, presence of symptoms or inducible myocardial ischemia. Multimodality imaging is crucial for diagnosis, management planning and follow up. Assessment of the risk of recurrent ventricular arrhythmias, despite full revascularization, should be performed in all adults with ALCAPA. Myocardial scar detected via late gadolinium enhancement represents a potential irreversible substrate for ventricular arrhythmias, and it provides additional information to evaluate indication of an ICD for secondary prevention.

## 1. Introduction

Coronary artery anomalies include morphological features seen in less than 1% of the general population and are classified into coronary origin, course and termination anomalies [1,2]. Embryologically, anomalies of coronary origin are due to either malrotation of the spiral septum dividing the truncus or malpositioning of the coronary buds [3]. Anomalous origin of a coronary artery from the pulmonary artery (ACAPA) is a rare congenital anomaly, which results in low oxygen levels, coronary steal syndrome and myocardial ischemia. ALCAPA can be associated with other congenital heart defects, such as ventricular septal defect, patent ductus arteriosus, tetralogy of Fallot, and aorto-pulmonary window [4]. The anomalous origin of the left coronary artery (LCA) from the pulmonary artery (PA) is known as ALCAPA or Bland-White-Garland syndrome, and it represents 0.25–0.5% of diagnosed congenital heart disease (CHD). In ALCAPA the left ventricular myocardium receives desaturated blood at low perfusion pressures from the PA with development of coronary collaterals, which usually appear dilated [2]. The pressure in the other coronary arteries such as the right coronary artery (RCA) exceeds LCA pressure and this eventually leads to blood flowing from the RCA to the LCA and into the PA, forming a left-to-right shunt. The anomalous origin of the RCA from the PA, known as ARCAPA, is less frequent and accounts for 0.002% of all CHD. Other variants of ACAPA include the origin of an accessory coronary artery from the PA and the origin of the entire coronary circulation from the PA [5]. ALCAPA is more common than ARCAPA because the left coronary bud in the embryo is located more closely to the pulmonary artery sinus compared to the right coronary bud [3].

Prior to birth, a patent ductus arteriosus determines equivalent pressures in the main PA and aorta, and there are relatively equivalent oxygen concentrations. Therefore, prenatally there is normal myocardial perfusion and no stimulus for collateral vessel formation. After birth, the ductus arteriosus closes and pulmonary vascular resistance and pulmonary artery pressure decrease; therefore, there is reversal of flow from the anomalous coronary and the left ventricular myocardium receives desaturated blood at low perfusion pressures. In the absence of an adequate collateral supply, these patients may develop myocardial ischemia, which may progress to myocardial infarction, left ventricle dilation, congestive heart failure (CHF, due to volume overload from the left-to-right shunt which determines increased LV preload), mitral regurgitation, arrhythmia and sudden cardiac death (SCD). Cardiac arrest may be due to primary ventricular fibrillation (VF) caused by reversible ischemia or could be due to scar-related (irreversible) re-entrant ventricular tachycardia (VT) degenerating into VF [6]. ARCAPA generally causes milder ischemia since the right ventricular demands are lower but may be less tolerated in the case of a dominant RCA system [7]. The majority of patients (90%) with ALCAPA, if not operated on, die within the first year of life [8], from ischemic cardiomyopathy and endocardial fibrosis due to reduced blood supply in LAD territory. There are only a few cases of ALCAPA diagnosed in adulthood, mainly in patients with an adequate collateral circulation that allows them to remain asymptomatic for many years.

## 2. Case Report

### Case Presentation

A 55-year-old Asian female presented with witnessed syncope while exercising at the gym. Pre-hospital cardiopulmonary resuscitation (CPR) was immediately initiated due to SCD. The patient’s rhythm was ventricular fibrillation, therefore, immediate defibrillation with two 200 J DC-shocks was delivered and intravenous epinephrine was administered, achieving return of spontaneous circulation (ROSC) after 8 min. After ROSC, an electrocardiogram (EKG) documented atrial fibrillation, treated with intravenous amiodarone administration, with return of sinus rhythm. The patients past medical history included a prior episode of SCD four years prior while exercising, with successful resuscitation. At the time, the EKG showed sinus rhythm with no signs of acute myocardial infarction. A head CT was negative for acute cardiovascular accident and cardiac enzymes were within normal limits. A 24-h Holter monitor did not reveal any arrhythmias and a transthoracic echocardiogram (TTE) documented normal left ventricular (LV) size, borderline concentric LV hypertrophy, normal LV systolic function with an ejection fraction (EF) of 60–65, normal right ventricular (RV) size and function, normal pulmonary artery systolic pressure, moderately dilated left atrium (LA) and no valvular abnormality. The patient was given an appointment with an electrophysiology cardiologist, but she decided not to follow up. Her family history included a first-degree relative (mother) that died at a young age (37 years old) for unknown reasons.

**Figure 1:**
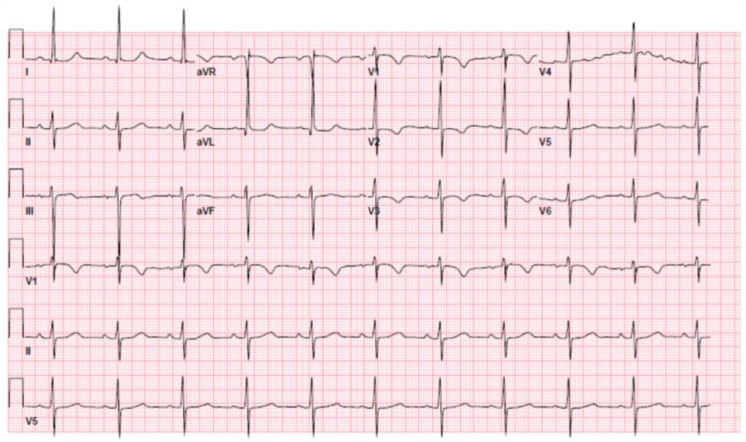
Electrocardiogram at arrival, documenting normal sinus rhythm, left anterior fascicular block and diffuse non-specific T wave abnormalities.

**Figure 2:**
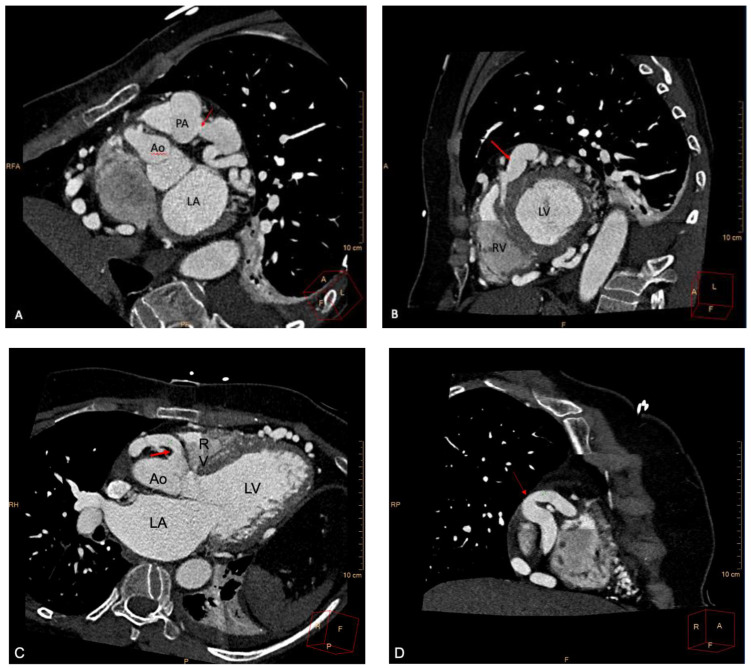
Coronary computed tomography angiography (CCTA) showed (**A**) anomalous origin of the left coronary artery (LCA, red arrow) from the main pulmonary artery (PA), (**B**) ectatic and tortuous LCA (red arrow), (**C**) normal origin of the right coronary artery (RCA, red arrow) from the right coronary sinus and (**D**) diffusely ectatic and tortuous RCA system (red arrow). Ao = Aorta, LA = left atrium, LV = left ventricle, RV = right ventricle.

At her current presentation, her vital signs were as follows: blood pressure 118/84 mmHg, heart rate 68 beats per minute, respiratory rate 20 breaths per minute, SpO2 94% on room air, body temperature 98.2 °F (36.8 °C). Physical examination documented clear lungs bilaterally, normal S1 with a long systolic murmur, no signs of trauma and no neurological deficits. The patient’s weight was 59 kg, her height 165 cm and her body mass index 21.7 kg/m^2^. She had no history of alcohol, illicit drug use or any home medications. Her laboratory evaluation revealed a hemoglobin of 10.7 g/dL, white blood cells of 6.0 K U/L, platelets 168 K U/L, sodium 139 mEq/L, potassium 3.8 mEq/L, bicarbonate 15 mEq/L, calcium 8.8 mg/dL, magnesium 1.9 mg/dL, creatinine 0.69 mg/dL, AST 101 U/L, ALT 160 U/L, troponin T 0.188 ng/mL (normal reference range < 0.010 ng/mL) and glucose 92 mg/dL. An EKG showed normal sinus rhythm at 64 beats per minute, left anterior fascicular block, normal atrioventricular conduction and non-specific diffuse T wave abnormalities (Figure 1). A TTE documented mild LV dilation, preserved LV systolic function without regional wall motion abnormalities, normal RV size and function, severely dilated LA, no valvular abnormality or pericardial effusion and, most interestingly, anomalous origin of the LCA from the main pulmonary artery (MPA), with retrograde, low-velocity flow into the leftward aspect of the MPA and a severely dilated proximal RCA with antegrade flow. Moreover, multiple collateral vessels were seen in the ventricular septum coursing posterior-anterior and right-left within the septum. Cardiac catheterization did not reveal any coronary artery stenosis but documented anomalous origin of the LCA from the MPA with retrograde filling. Coronary computed tomography angiography (CCTA) confirmed an anomalous LCA origin from the left aspect of the MPA with a diffusely ectatic/aneurysmal (measuring up to 2.7 cm) and tortuous left coronary artery system (Figure 2a,b; Supplementary Video S1). CCTA also documented a retrograde filling of the distal left anterior descending (LAD) artery at the LV apex via the right coronary artery (RCA)/right posterior descending artery, normal origin of the RCA from the right coronary cusp (Figure 2c; Supplementary Video S1) with a diffusely ectatic and tortuous RCA system (Figure 2d; Supplementary Video S1), no definitive evidence of coronary atherosclerosis or stenosis, a mildly dilated LV and a small left pleural effusion with subjacent atelectasis/consolidation. The patient’s calcium score was 0.

**Figure 3:**
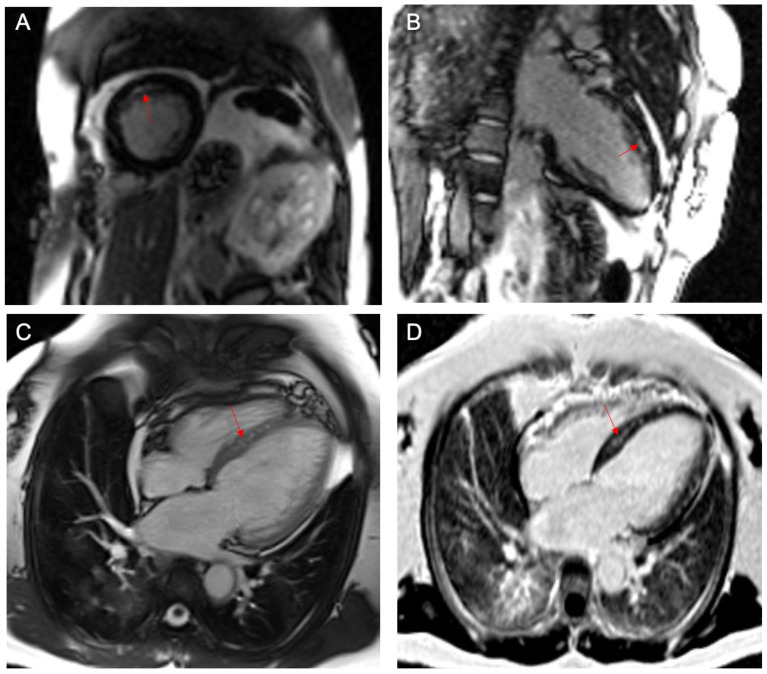
Cardiac magnetic resonance (CMR) with gadolinium demonstrated subendocardial enhancement of the basal-to-mid anterior wall (**A**,**B**, red arrows). In addition, a coronary perforator in the interventricular septum was documented pre-contrast (**C**, red arrow) and post-contrast injection (**D**, red arrow).

Stress myocardial perfusion imaging (MPI) was abnormal and consistent with moderate antero-septal ischemia with preserved LVEF. Subsequently, the patient underwent a cardiac magnetic resonance (CMR) that documented mild LV dilatation, normal RV size, and normal biventricular systolic function and subendocardial late gadolinium enhancement (LGE) of the basal-to-mid anterior wall (Figure 3). A magnetic resonance angiogram (MRA) demonstrated the ALCAPA findings.

**Figure 4:**
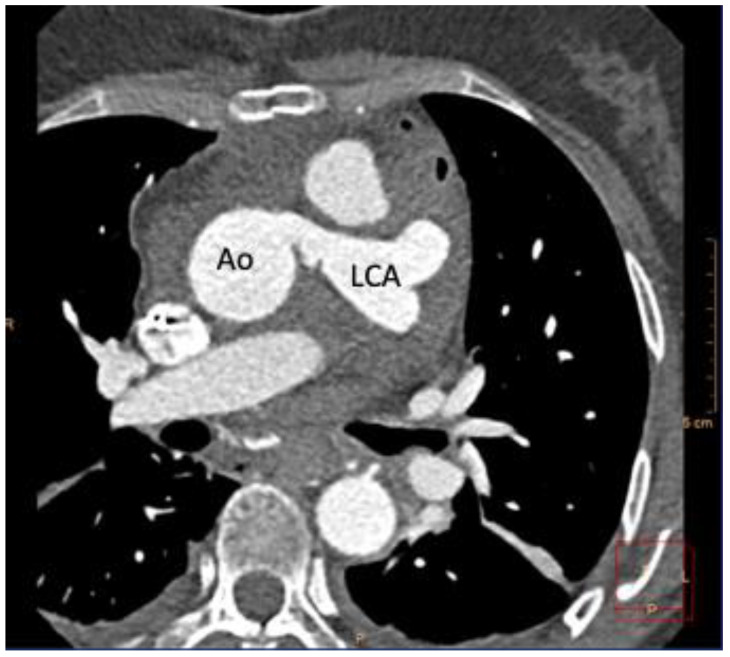
Post-operative coronary computed angiography (CCTA) displaying anastomosis via 10 mm Hemashield graft between the Aorta (Ao) and left coronary artery (LCA).

In consideration of the two episodes of cardiac arrest, the family history, the ALCAPA diagnosis and the myocardial scarring documented via LGE, an implantable cardioverter defibrillator (ICD) was placed for secondary prevention. The patient was started on metoprolol tartrate 25 mg bid, atorvastatin 40 mg per day and aspirin 81 mg per day. Surgical ALCAPA repair was planned. A transesophageal echocardiogram (TEE) was performed, revealing the dilated proximal coronary arteries, retrograde low-velocity flow into the leftward aspect of the MPA, proximal RCA with antegrade flow (Supplementary Video S2), LMCA and LAD with retrograde low-velocity flow into the leftward aspect of the MPA, multiple collateral vessels in the ventricular septum coursing posterior-anterior and right-to-left within the septum and fistulous communication between these collaterals and the RV cavity. The patient underwent successful ALCAPA repair with direct reimplantation of the LCA into the aorta, with a 10 mm Hemashield interposition graft between the LCA and aorta and a 28 mm Hemashield interposition graft between the proximal main PA and the distal main PA, without any intra- or peri-procedural complications. A TEE after surgery documented difficulty in visualizing the interposition graft from the aortic sinus to the LCA, but antegrade flow was seen in the LCA. Color-flow mapping at low Nyquist limit over the RV and ventricular septum revealed significantly less coronary-RV flow compared to the pre-operative TEE. CCTA after surgery documented marked diffuse dilatation and tortuosity of the coronary arteries consistent with a history of ALCAPA, the LCA connecting to the left aspect of the tubular ascending aorta, with patent interposition grafts between the LCA and ascending aorta (Figure 4), and in the main PA, normal left ventricular size, low normal LV systolic function (EF = 51%), small pericardial effusion with foci of pneumopericardium consistent with recent surgery and small bilateral pleural effusions (left > right) with left lower lobe atelectasis. A TTE performed at the 1-month follow-up documented mildly dilated LV, low normal LV systolic function, normal RV size and function, moderately dilated LA, diffusely dilated proximal coronary arteries, severely dilated proximal RCA with antegrade flow, interposition graft from aortic sinus to LCA with antegrade flow in LCA, small pericardial effusion (maximum 8 mm) and no pleural effusion. At the three-month follow-up, the patient remained asymptomatic and had no surgical complications and had returned to mild levels of activity.

## 3. Discussion

ALCAPA is a very uncommon congenital coronary artery anomaly, most commonly presenting in early infancy. Late adult presentation of ALCAPA syndrome is extremely rare. Presenting features of ALCAPA may include a continuous murmur at the left parasternal border, chest pain, myocardial infarction, LV dysfunction, severe valvular disease such as mitral regurgitation, arrhythmia, dyspnea, palpitations, cyanosis, syncope and, rarely, sudden cardiac death.

Coronary artery anomalies can be lethal mostly during or shortly after strenuous physical activity [9]. Coronary artery anomalies are responsible for 11% of SCD cases in exercising individuals [10] and 19% in competitive athletes [11]. Unlike ALCAPA, ARCAPA rarely leads to SCD [6], although there are some case reports. Only a very few case reports of out-of-hospital cardiac arrest presentation in an adult with ALCAPA were previously described [6,12,13,14,15,16].

According to ACC/AHA 2018 Guidelines for the management of adults with CHD, it is recommended to perform anomalous coronary artery evaluation via coronary angiography, using catheterization, CT or MRI. CT is generally preferred because of its superior spatial and temporal resolution [9]. Diagnostic tests important in ALCAPA include an EKG, which may reveal ischemic signs at rest, with Q waves and ST changes especially in the anterior and lateral leads, and also during exercise testing [4]. A TTE shows generally retrograde flow from the LCA to the PA and a dilated proximal RCA; frequent findings include LV dilation, wall motion abnormalities and mitral regurgitation, which can be functional (secondary to LV dilation) or related to papillary muscle ischemia and fibrosis. In this anomaly, LMCA territory function often requires extensive collateral circulation from the RCA, which often is dilated [2]. The collateral circulation between the RCA and LCA is on the epicardial level and via septal perforators, which were both present in our patient. The high blood flow determines dilation of the epicardial arteries, due to local endothelial-derived shear stimuli [17]. A TEE is useful to further delineate the anatomy of ALCAPA and of the coronary flow patterns [12]. However, cardiac computed tomography and CMR are generally gold-standard techniques which confirm the definitive diagnosis, with direct visualization of the vascular anatomy with 3D reconstruction of the coronary tree. CMR is also useful to evaluate though LGE the presence of myocardial scar, which is important to assess the risk of recurrent ventricular arrhythmias and the indication for an ICD. Myocardial perfusion imaging may be abnormal and document signs of ischemia. Cardiac catheterization may provide information on the extent of collaterals, the entity of the left-to-right shunt and the end-diastolic pressures [4].

Surgical correction provides symptomatic and prognostic benefit and is recommended (class I) in all cases of anomalous origin of the left circumflex artery from the pulmonary artery, regardless of age, symptomatic status or presence of inducible myocardial ischemia, in order to restore normal coronary flow and avoid the progression of ischemia, fibrosis, CHF, arrhythmia and SCD. In ARCAPA, surgery is recommended (class I) in symptomatic adults in which symptoms are attributable to the coronary anomaly, and it is reasonable (class IIa) in asymptomatic adults with ventricular dysfunction or myocardial ischemia attributable to the coronary anomaly [9,18]. In ALCAPA, re-implantation of the LCA in the correct aortic sinus, with or without an interposition graft, is the preferred choice. Alternatively, an anomalous LCA is completely ligated or closed at the level of the PA and a left internal mammary artery or a saphenous vein graft is placed, usually anastomosed to the LAD [9,19]. In ARCAPA cases, surgery can include reimplantation of the RCA into the aorta, with or without an interposition graft, or ligation or closure of the RCA at the level of the PA with a coronary artery bypass graft using the right internal mammary artery, usually anastomosed to the RCA or posterior descending coronary artery [9]. A CABG with closure of ALCAPA/ARCAPA should be reserved only for those patients in which coronary transfer is not feasible [18]. In patients with significant mitral regurgitation, mitral valve repair may also be necessary. Recently, a successful case of conservative management of ALCAPA was reported, regarding a woman who was diagnosed with ALCAPA in her 8th decade of life, during elective coronary angiography for significant ST-depression at stress test. The patient was asymptomatic, had good collateral circulation and after appropriate discussion with the heart team, a decision was made for conservative medical management; at the three-year follow-up the patient had experienced no cardiovascular complication [20].

An ICD for secondary prevention is indicated in sudden cardiac arrest survivors without a reversible cause [21]. The event is called sudden cardiac arrest or aborted SCD if an intervention (such as defibrillation) or spontaneous reversion restore circulation, while it is called SCD if the patient dies [22]. Restoration of a dual coronary system will prevent further ischemia and ventricular arrhythmias of acute ischemic origin, but the scarred anatomical substrate will not be altered after revascularization. Before corrective surgery, patients with ALCAPA often present a severely compromised LV function and evidence of scarring at LGE, but in long-term follow-up, scar tissue is relatively scarce [23]. An increased extent of LGE with >50% transmural involvement is an independent predictor for recurrence of ventricular arrhythmias. LGE-CMR should be incorporated in a patient-guided risk assessment for survivors of sudden cardiac arrest, since it provides additional information to evaluate indication of an ICD in secondary prevention [24]. An ICD implantation should be considered when myocardial scar is detected, despite full revascularization and preserved EF, since it represents an irreversible potential substrate for ventricular arrhythmias and cardiac arrest [6].

## 4. Conclusions

Although many congenital coronary abnormalities have a benign outcome, natural history of ALCAPA shows a poor outcome in untreated patients [25]. ALCAPA is a rare coronary anomaly that usually manifests in the first month of life. Very rarely, ALCAPA syndrome may present in late adulthood, and this can be attributed to the presence of a well-collateralized and pressurized coronary network system from the RCA to the LCA. Clinical presentation is variable and includes angina, myocardial infarction, heart failure, cardiac arrest and SCD.

Multimodality imaging is crucial for the initial diagnosis, therapeutic management and follow up. Surgical repair provides symptomatic and prognostic benefit, and it is recommended in all cases of ALCAPA, regardless of age, symptomatic status or presence of inducible myocardial ischemia. Assessment of the risk of recurrent ventricular arrhythmias despite full revascularization should be performed in all adults with ALCAPA. Myocardial scar detected via LGE represents a potential irreversible substrate for ventricular arrhythmias and an ICD in secondary prevention should be considered.

## Data Availability

Not applicable.

## Supplementary Materials

The following are available online at https://www.mdpi.com/article/10.3390/ijerph19031554/s1, Supplementary Video S1: Coronary computed tomography angiography (CCTA) showed anomalous origin of the left coronary artery (LCA) from the main pulmonary artery, with ectatic and tortuous LCA, and normal origin of the right coronary artery (RCA) from the right coronary sinus, with diffusely ectatic and tortuous RCA system. Supplementary Video S2: Transesophageal echocardiogram (TEE) with color-Doppler showed dilated proximal RCA with antegrade flow.
